# Supplementary material for: Multi‐dynamic deep image prior for cardiac MRI
Source: Magn Reson Med. 2025 Jul 22;94(6):2668–79. doi: 10.1002/mrm.70000 (PMC12501714; doi:10.1002/mrm.70000)
Supplement: Supplementary file 1 — Data S1. Supporting Information. [file MRM-94-2668-s001.zip › M-DIP_Supporting_Documents.pdf]

Multi-dynamic deep image prior for cardiac MRI
Supporting information

Marc Vornehm, et al.

Table S1: Reconstruction hyperparameters. M-DIP parameters: Number of dictionary elements (L), regularization weights for spatial and temporal smoothness of the deformation fields (λ_s and λ_f, respectively), initial noise regularization level (σ_0), initial learning rates for static and dynamic model components (η_s and η_f, respectively), number of training iterations (N_iter), and number of iterations after which deformation fields are generated (N_def). LR-DIP parameters: Rank of the low-rank system (k_lr) and depth of the spatial and temporal basis U-Nets (d_S and d_T, respectively). L+S parameters: Regularization parameters for the low-rank (λ_L) and sparse (λ_S) component, respectively.

	M-DIP								LR-DIP			L+S	
Dataset	L	λ_s	λ_f	σ_0	η_s	η_f	N_iter	N_def	k_lr	d_S	d_T	λ_L	λ_S
Phantom	16	0.02	0.02	0.01	$1 \cdot 10^{-3}$	$1 \cdot 10^{-3}$	10 000	0	64	5	5	0.5	0.05
Cine	16	0.1	0.05	0.05	$1 \cdot 10^{-3}$	$1 \cdot 10^{-3}$	10 000	0	64	5	5	0.5	0.05
LGE	8	0.1	0	0.1	$5 \cdot 10^{-4}$	$1 \cdot 10^{-3}$	10 000	0	12	5	4	0.01	0.1
Perfusion	24	0.2	0.02	0.05	$3 \cdot 10^{-4}$	$6 \cdot 10^{-3}$	8 000	1 000	24	5	5	0.01	0.5

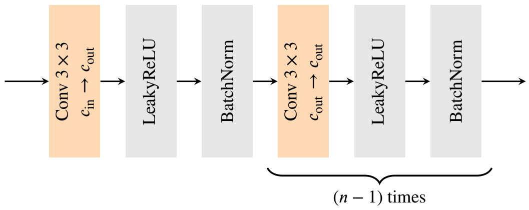

(a) Convolutional block ConvBlock$(n, c_{\text{in}}, c_{\text{out}})$. n, c_{in}, and c_{out} are the number of convolutional layers, input channels, and output channels, respectively.

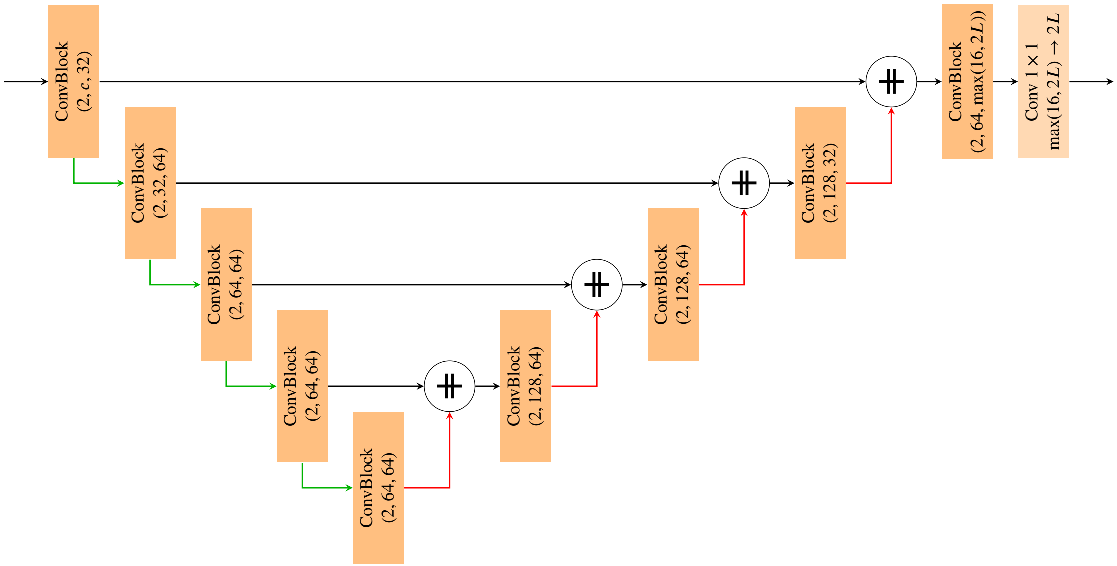

(b) Spatial dictionary generator $\mathcal{G}_{\boldsymbol{\theta}}$. Green arrows denote average pooling with kernel size 2×2 and red arrows denote interpolation by a factor of two. $\mathbin{+\!\!+}$ denotes concatenation along the channel dimension.

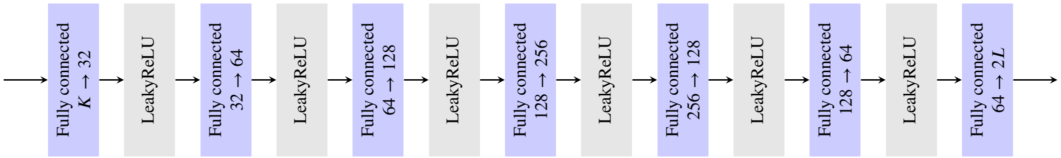

(c) Temporal weights generator $\mathcal{G}_{\boldsymbol{\zeta}}$.

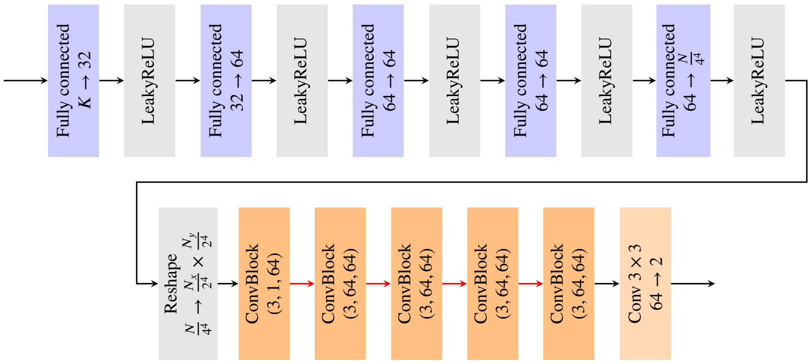

(d) Deformation field generator $\mathcal{G}_{\boldsymbol{\psi}}$. N_x and N_y are the number of image pixels along the spatial dimensions.

Figure S1: Detailed network architectures of (a) convolutional blocks, (b) spatial dictionary generator $\mathcal{G}_{\boldsymbol{\theta}}$, (c) temporal weights generator $\mathcal{G}_{\boldsymbol{\zeta}}$, and (d) deformation field generator $\mathcal{G}_{\boldsymbol{\psi}}$.
